# Nontarget catches of traps with chemical lures may refer to the flower‐visitation, probable pollination, and feeding of bush crickets (Ensifera: Tettigoniidae)

**DOI:** 10.1002/ece3.10249

**Published:** 2023-07-04

**Authors:** Antal Nagy, Aletta Ősz, Miklós Tóth, István András Rácz, Szilvia Kovács, Szabolcs Szanyi

**Affiliations:** ^1^ Faculty of Agricultural and Food Sciences and Environmental Management, Institute of Plant Protection University of Debrecen Debrecen Hungary; ^2^ Plant Protection Institute, Centre for Agricultural Research ELKH Budapest Hungary; ^3^ Department of Evolutionary Zoology and Human Biology University of Debrecen Debrecen Hungary; ^4^ Department of Applied Plant Biology, Institute of Crop Sciences, Faculty of Agricultural and Food Sciences and Environmental Management University of Debrecen Debrecen Hungary

**Keywords:** floriphagous, floriphilic, orthoptera, pollination, volatile traps

## Abstract

The diurnal bees, lepidopterans, and other pollinators are among the most studied flower‐visiting insect taxa. They mostly play distinct functions in temperate grasslands and ecotones of grassland‐forest mosaics (such as in forest steppes). Although orthopterans are widely distributed in these habitats, however, their flower visitation is nearly unknown, especially in the temperate zone. During the development of traps with chemical lures to catch Lepidoptera pests, large numbers of Orthoptera were caught that provide a chance for studying the flower visitation and odor and indirectly the host plant preference of seven temperate zone Tettigoniidae species. Data on the attractivity of isoamyl alcohol‐based semisynthetic lures for *Meconema thalassinum* and efficiency of phenylacetaldehyde‐based lures on *Leptophyes albovittata* and *Phaneroptera falcata* were reported for the first time. Additionally, analysis of nature photos collected from internet sources, as part of a passive citizen science also supports the revealed preference of these species. Based on photos, the studied orthopterans mainly visit Asteraceae species including the most preferred *Tanacetum vulgare*, *Pulicaria dysenterica*, *Achillea millefolium*, *Solidago canadensis*, and *Centaurea scabiosa*. Based on catches of volatile traps, the first data were recorded on the attractivity of phenylacetaldehyde‐ and isoamyl alcohol‐based lures on three temperate zone Orthoptera species. Results of a passive citizen science study strengthen these results that may increase the knowledge on the host plant and habitat preference of Orthoptera species.

## Graphical abstract

The flower visitation and host plant preference of seven temperate zone Tettigoniidae species were studied during the development of traps with chemical lures. Large numbers of Orthoptera were caught, providing an opportunity for analysis. Based on data from chemical lures and nature photos, the studied Orthoptera species mainly visit Asteraceae species, including the most preferred *Tanacetum vulgare*, *Pulicaria dysenterica*, *Achillea millefolium*, *Solidago canadensis*, and *Centaureae scabiosa*.

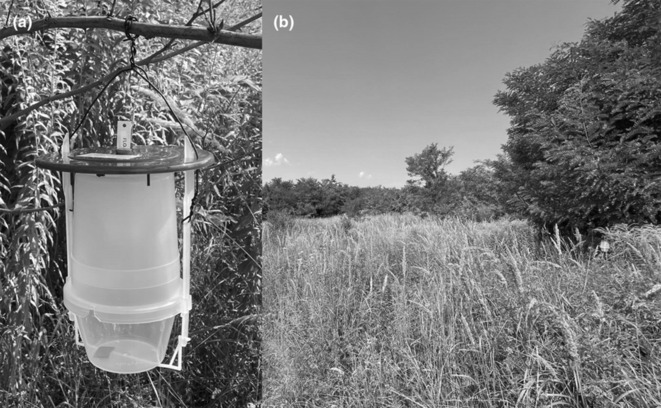

## 1 INTRODUCTION

Pollination is one of the basic requirements of the sexual reproduction of angiosperm plants. The delivery of pollen can happen in several ways, but the role of pollinators, especially insects, is outstanding (Katumo et al., 2022; Kevan, 1999). Beyond pollination, there are many aspects of flower visitation in insect‐plant interactions since plants, especially flowers, may provide food source, habitat, shelter, and site for reproduction and oviposition for insects.

Thousands of insects visit flowers, and many of them feed mainly on them (Kondo et al., 2016; Rentz & Clyne, 1983). Florivores feed on pollen and different parts of the flowers directly (e.g. Nitidulidae, the apple blossom weevil *Anthonomus pomorum* (L.), etc.; Tan et al., 2017; Wardhaugh, 2015) or use flowers as nectar sources (e.g. intensively feeding lepidopterans: Noctuidae, Nymphalidae, etc.; Grundel et al., 2000) attracted by the odor of their host plants (Blackiston et al., 2011; Weiss & Papaj, 2003).

Among the flower‐visiting insects, the diurnal bees, lepidopterans, and other pollinators are relatively well studied (Hoehn et al., 2008; Kevan et al., 1990). Nevertheless, other less‐known taxa such as beetles, orthopterans, hoverflies, and nocturnal lepidopterans (Breadmore & Kirk, 1998; McCall & Irwin, 2006) also may have essential role in the pollination, and have direct or indirect effects on flower adaptation and community dynamics (Frame, 2003; Krupnick et al., 1999; McCall & Irwin, 2006). Many of them are rather known as pests e.g. some Orthoptera, and many noctuids (Haile et al., 2021). The species of the Orthoptera order can provide mutualistic ecological services including seed dispersal (Duthie et al., 2006; Suetsugu, 2018), but they are rarely considered capable of pollination (Micheneau et al., 2010).

There are more than 28,500 known Orthoptera species worldwide (Cigliano et al., 2023; Myers et al., 2000; Tan et al., 2017). They occur in grasslands and ecotones of grassland‐forest mosaics (e.g. in forest steppes) in the temperate zone (Nagy et al., 2019) and species‐rich assemblages inhabit woodlands, especially in tropical and subtropical areas (Tan et al., 2017). They may be herbivores (e.g. species of Caelifera order and many members of Tettigoniidae family (Ensifera)), predators (as some Tettigoniidae e.g. *Saga* spp.), and omnivores such as the most species of Ensifera order including species of Grylloidea and many other groups (Sanam et al., 2021; Xu et al., 2013). Due to their large biomass, herbivore species play an important role in the food webs of grasslands and beyond that, some flower‐visiting species are described as pollinators.

Orthoptera pollination is a little studied phenomenon, and we have valid data on it mainly from the tropical regions, although the connection between orthopterans and flowers originated from the late Jurassic and early Cretaceous periods (Song et al., 2015). According to Krenn et al. (2016) obligatory flower‐visiting orthopterans are rare, and all belong to the Ensifera order. Some Australian species of the Zaprochilinae subfamily feed on flowers but do not pollinate them (Kevan & Baker, 1983). Contrarily, some neotropical crickets and endemic wetas of New Zealand (species of Anostostomatidae and Rhaphidophoridae families) are probable pollinators. Later Lord et al. (2013) described *Notoplectron campbellensis* Rich. (Rhaphidophoridae) as a night‐active pollinator of *Veronica benthamii* Hook., *Anisotome latifolia* Hook. and *Bulbinella rossii* Hook. Hugel et al. (2010) described *Glomeremus orchidophilus* (Hugel), a formerly unknown species from the island of Mauritius and Reunion (Mascarene Island in the Indian Ocean), which is a single pollinator of the *Angraecum cadetii* Bos. orchid (Micheneau et al., 2010). The most intensive studies on Orthoptera pollination were carried out in the Indo‐Malayan region during recent years (Tan et al., 2019; Tan & Tan, 2017) when two main types of flower‐visiting orthopterans were described. One of them is the group of floriphilic species (which prefer flowers as food sources) containing species of the Phaneropterinae subfamily (e.g *Phaneroptera brevis* Serville). *P. brevis* adults and nymphs are commonly seen feeding on the inflorescences of *Bidens pilosa* Lin. (Asteraceae), a plant native to tropical America and naturalized throughout disturbed areas of warm temperate and tropical regions of the World (Strother & Weedon, 2006). The other group consist of opportunist polyphagous species, which are florivores or facultative florivores. Some of them are known pests (e.g. *Valanga nigricornis* Bur. and *Xenocatantops humilis* Ser.), while other Tettigoniidae species (e.g. some *Conocephalus* spp. and *Tremellia timah* Gor.‐Tan) mainly feed on leaves of bushes and trees and feeding on flowers occasionally (Higginson et al., 2015).

The flower visitation of temperate zone orthopterans is nearly unknown and there is no published data on this topic, and the host plant preference of the species is also mostly unknown. Using data collected by traps baited with chemical lures designed for plant protection monitoring (Nagy et al., 2022; Szanyi et al., 2019) and analyzing nature photos of the potential flower‐visiting species here we provide the first data on the flower‐visiting, probable pollination, and host plant preferences of seven Tettigoniidae species living in Central Europe. Beyond the scientific novelty, our results help to protect and manage the general pollinator communities of the temperate zone.

## 2 MATERIALS AND METHODS

### 2.1 Sampling area

**FIGURE 1:**
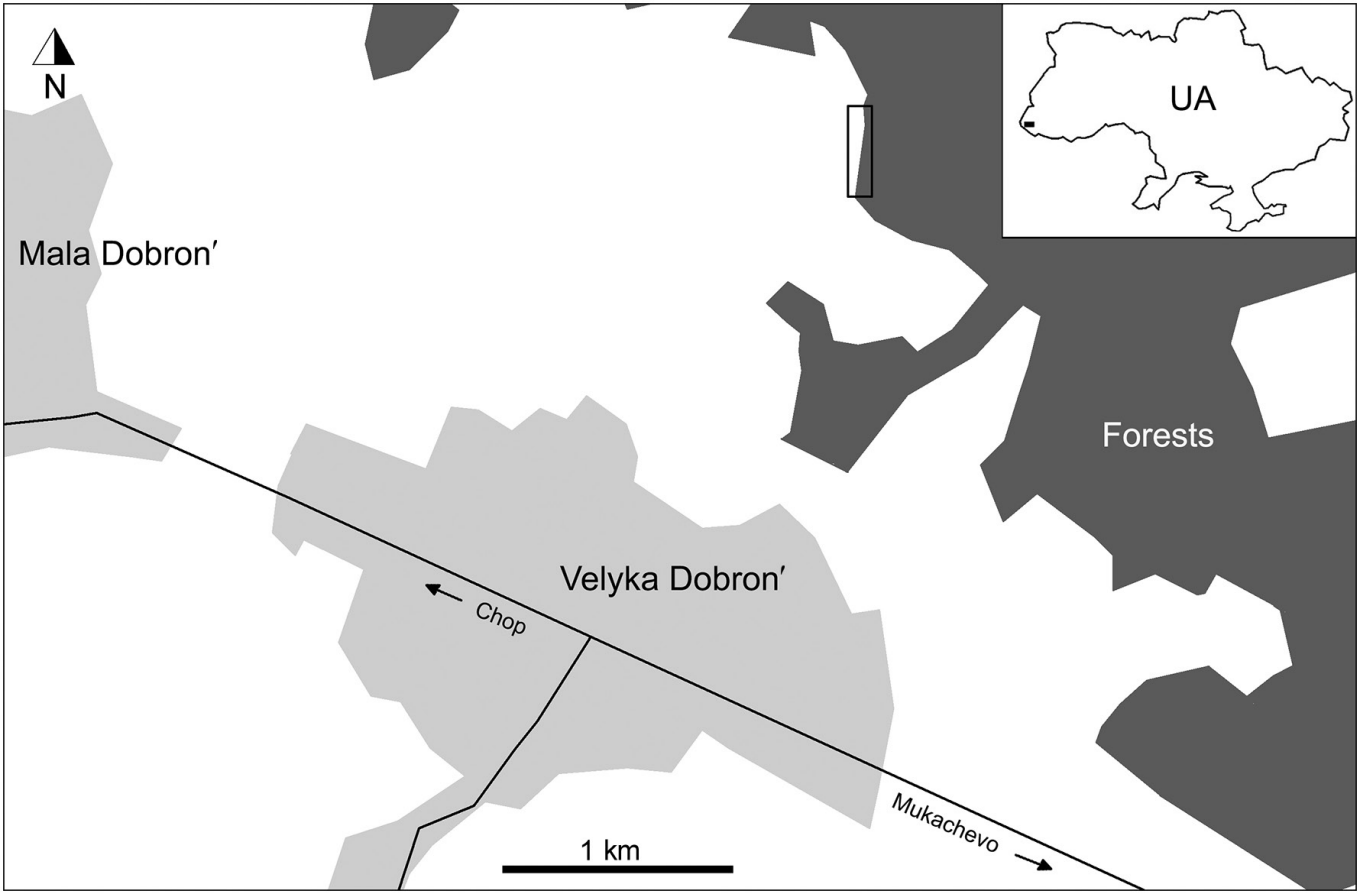
Location of the sampling site near Velyka Dobron' village at a forest margin (empty black rectangular) in Transcarpathia (West Ukraine).

**FIGURE 2:**
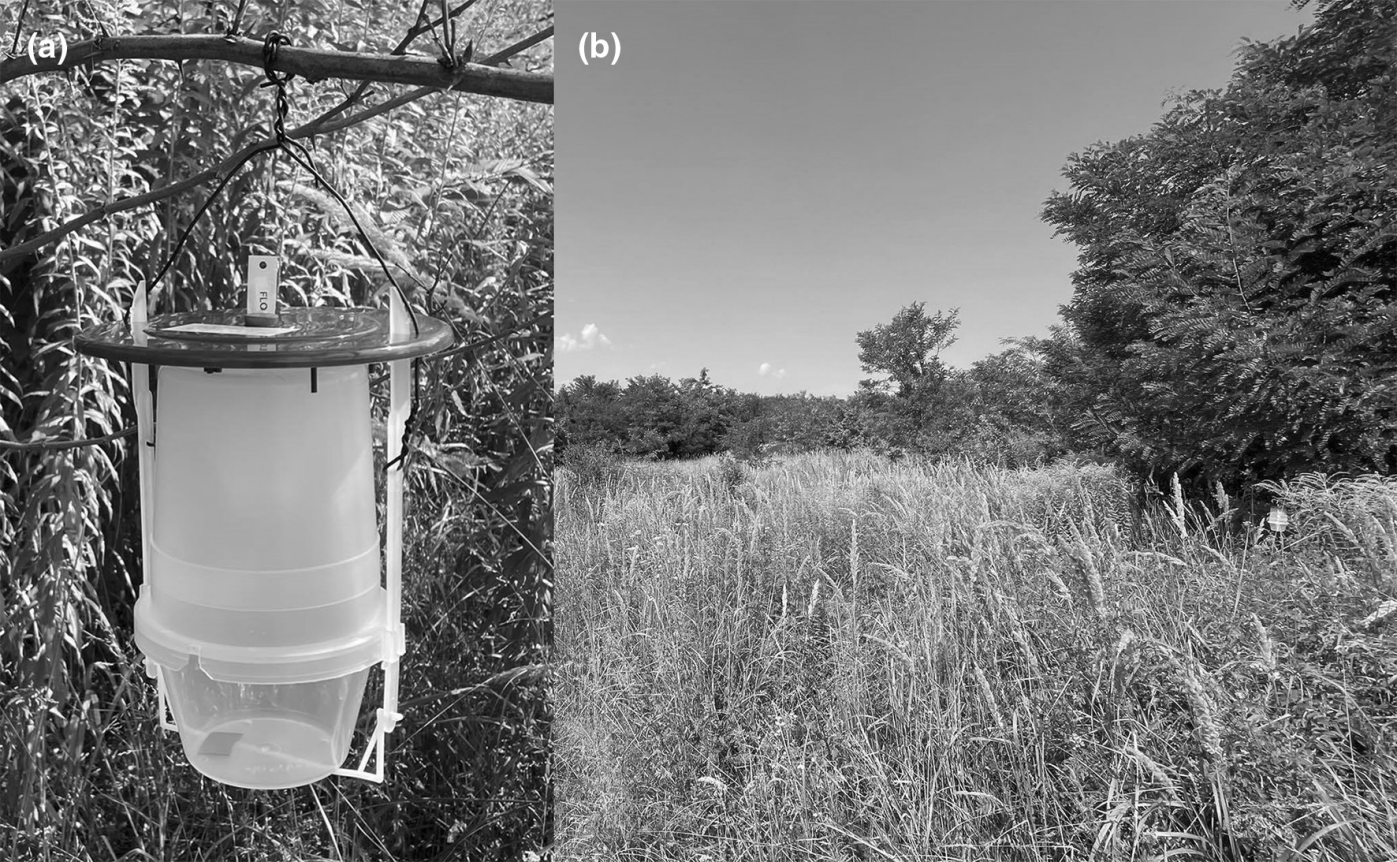
VARL+ funnel trap (CSALOMON®) (a) on the forest margin (b) near Velyka Dobron' village.

Trap samplings were carried out in the Velyka Dobron' Forest close to the Velyka Dobron' Game Reserve, situated on the margin of the former Szernye Marsh in the Ukrainian part of the Bereg Region (Transcarpathia, West Ukraine; GPS: N48.4338°, E22.4041°; Figures 1 and 2b). Although the ancient flora and vegetation of the peatland were extremely rich and valuable (Boros, 1964; Simon, 1952), the most important relict habitats and species have become extinct after water regulation in the 19th and 20th centuries. The area is recently dominated by secondary habitats with some fragments of the original wetland and forest vegetation. In the region hardwood gallery forest is one of the most important and valuable habitat types with closed canopy formed by *Quercus robur*, *Fraxinus angustifolia* subsp. *pannonica*, *Ulmus laevis*, *Populus canescens*, *Frangula alnus*, etc. The other one is lowland pedunculate oak‐hornbeam forest rich in geophytic species (*Scilla drunensis*, *Anemone nemorosa*, *A. ranunculoides*, *Isopyrum thalictroides*, etc.) and dominated by *Q. robur* and *Carpinus betulus*. Other components of the natural and semi‐natural vegetation are the more xerophilous silver lime (*Tilia tomentosa*)‐oak forests and forest fringes, the tall herbs of forest fringes, the humid forest clearings, and willow scrubs. The reserve is surrounded by extended agricultural lands and dissected by drainage channels of the former peatland (Szanyi et al., 2021). The most valuable grasslands are the remains of humid meadows maintaining a large amount of the former species‐rich assemblages indicated by orthopterans (Szanyi et al., 2017).

The Orthoptera fauna of the Bereg Region was formerly discussed considering both the Hungarian and Ukrainian parts of the region (Nagy et al., 2015). The assemblages of the most common habitat types were also described by Szanyi et al. (2017).

### 2.2 Sampling with traps

The surveys were carried out from 20th July to 19th October 2014, and from 12th July to 25th October 2015. CSALOMON® VARL+ funnel traps (www.csalomontraps.com; Figure 2a) were placed in margin of the Velyka Dobron' Forest. In the experiments, two synthetic phenylacetaldehyde‐based (PHEN1 and PHEN2) and a semisynthetic bisexual isoamyl alcohol‐based lure (SBL, see also e.g. Szanyi et al., 2022) were used parallelly with unbaited control traps (UNB).

The semi‐synthetic baits were made from 4 mL polypropylene tubes containing dental rolls soaked in a scent made of isoamyl alcohol, acetic acid, and red wine (1:1:1, 3 mL). The bait mixture evaporated through a 4 mm diameter opening (Tóth et al., 2015). The SBL lure imitates the odor of tree saps containing acetic acid as a decomposition product of sugars and potentially attracts species living in arboreal habitats.

In 2014, two separate polyethylene bag (PEbag) dispensers were used to bait PHEN lure traps (Tóth et al., 2015). In these dispensers, a dental roll was included in 1.5 × 1.5 cm polyethylene bags. One of the dispensers contained mixture of phenylacetaldehyde and (*E*)‐anethol (1:1) (PHEN1), while the other held phenylacetaldehyde, eugenol, and benzyl acetate (1:1:1) (PHEN2). In 2015, only the bait PHEN2 was used. The compounds evaporated through the wall of the 0.2 mm thick PEbag. PHEN lures contain compounds common in flower scents and they are potentially attractive for flower‐visiting insects.

The Vaportape® II pesticide strip, designed particularly for insect traps, was used to kill the trapped insects. The tested trap types (SBL, PHEN and UNB) were used in four repetitions, thus 12 (4 × 3) traps were placed on trees 20 m apart, at 1.8–2.0 m height. The traps were checked and emptied once a week and were also rotated weekly to reduce the bias caused by the location.

Since the tested volatiles are not species specific they attracted a wide range non‐target taxa beyond the targeted Lepidoptera pests. In the caught material there were many non‐target Lepidoptera (Nagy et al., 2015) and Diptera species (Katona et al., 2020). Bumblebees (Hymenoptera: Apidae) containing new taxa for the studied area, and some orthopterans were also caught.

Orthopterans were identified at species level based on the keys of Harz (1957, 1975, 1960), and the number of specimens caught was determined for each sample. Considering the nomenclature of the species the Orthoptera Species File online database was followed (Cigliano et al., 2023).

### 2.3 Photo collection and processing

In the case of species attracted by volatile traps, a passive citizen science study was carried out. Photos of flower visitations of the species were collected using Google search following the study of Van Zandt et al. (2019) who studied flower‐visiting of nocturnal lepidopterans based on pictures freely available on the internet. During the search, the scientific name of a given Orthoptera species was used alone and in combinations with “flower” and “on flower” words. Pictures of flower‐visiting orthopterans were separated and the plants were identified at species level, or if it was not possible at family level. Identification was carried out based on the keys of Király (2009).

### 2.4 Data analysis

The attractivity of the tested baits was compared using the total number and the mean number of caught individuals (individuals/trap/sample). The assumptions of parametric tests were tested with Q‐Q plots (normality) and Levene‐test (homogeneity of variances). Since our data did not fulfill these assumptions, the nonparametric Kruskall–Wallis test was used. If it showed significant differences, treatments were compared with Mann–Whitney *U*‐test.

During the photo analysis, the flower visitation of a given Orthoptera species was characterized by the ratio of photos showing it on a flower. The host plant preferences of the species were characterized by the number and ratio of plant taxa (family and species) appeared in the photos. The chemical characteristics of plant species were described based on the pherobase.com database.

## 3 RESULTS

### 3.1 Attractivity of lures

During the 2‐year sampling, 327 Tettigoniidae (Orthoptera) specimens belonging to seven species were caught by the baited traps: *Phaneroptera falcata* (Poda), *Leptophyes albovittata* (Kollar), *Conocephalus fuscus* (Fabricius), *Ruspolia nitidula* (Scopoli), *Tettigonia viridissima* (Linnaeus), *Pholidoptera griseoaptera* (De Geer), *Meconema thalassinum* (De Geer).

In the two studied years (2014 and 2015) *P. falcata*, *R. nitidula*, *M. thalassinum*, *L. albovittata* and *C. fuscus* showed enough high abundances for statistical analysis. Although *T. viridissima* catches were insufficient for statistical analysis it was mainly caught by traps baited with SBL lures, while *P. griseoaptera* had no clear preference (Table 1).

**TABLE 1:** Number of caught orthopterans by different trap types in the 2 years of the samplings carried out in the Velyka Dobron' Forests (West Ukraine).

	2014				2015				Sum
	PHEN	SBL	UNB	Sum	PHEN	SBL	UNB	Sum	
*Phaneroptera falcata*	44	3	16	63	44	1	10	55	118
*Meconema thalassinum*	1	18	8	27	6	25	6	37	64
*Conocephalus fuscus*	0	12	7	19	1	20	7	28	47
*Ruspolia nitidula*	9	18	5	32	2	3	1	6	38
*Leptophyes albovittata*	19	0	0	19	11	0	1	12	31
*Tettigonia viridissima*	0	6	3	9	1	9	4	14	23
*Pholidoptera griseoaptera*	0	0	0	0	2	3	1	6	6
Total number of individuals				169				158	327

**FIGURE 3:**
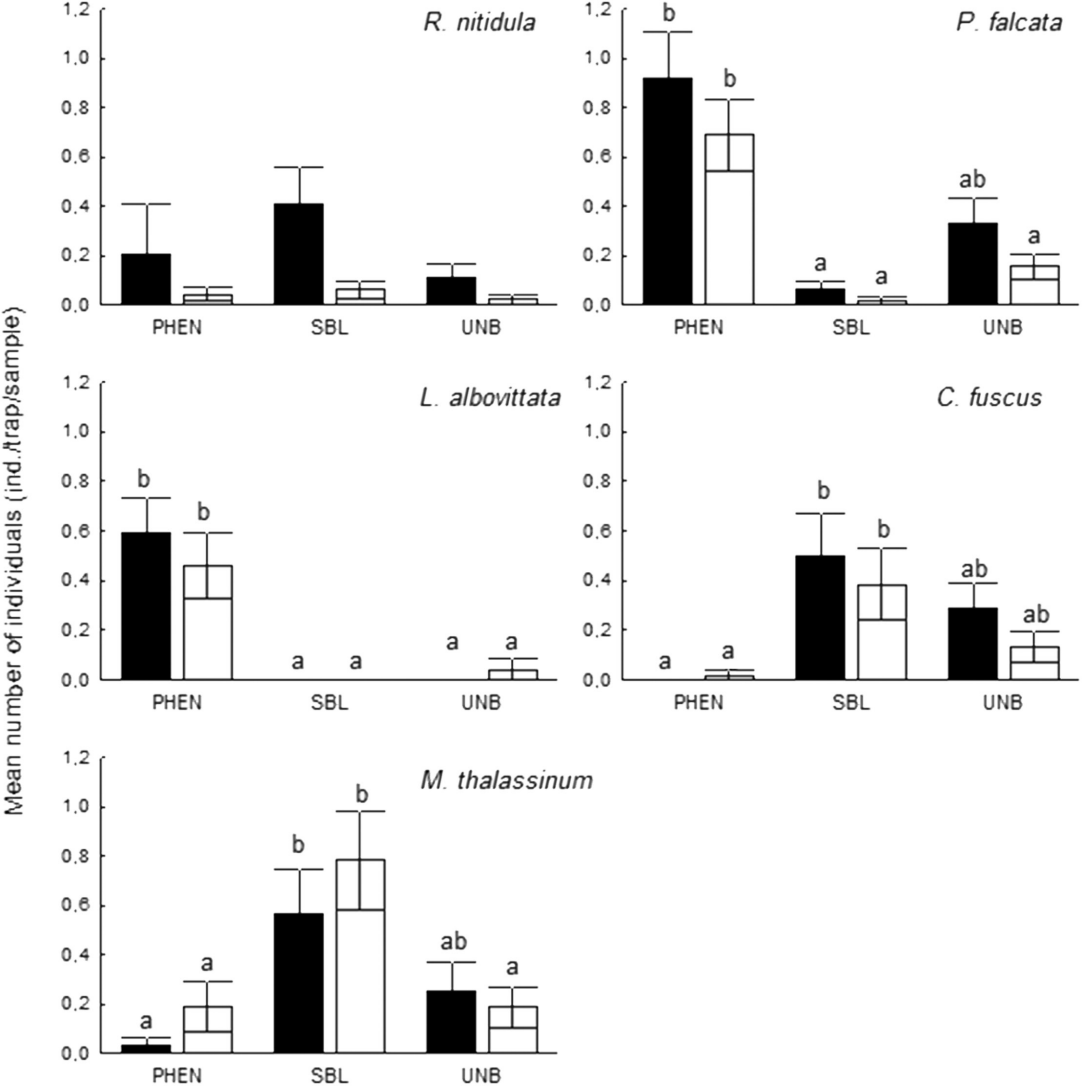
Mean number of caught Orthoptera specimens (individuals/trap/sample) attracted by different baits in 2014 (black bars) and 2015 (white bars). Letters show significant differences based on the Mann–Whitney *U* test (*p* < .05).

*Phaneroptera falcata* living in bushy habitats and forest edges was the most abundant species in both studied years (Table 1). In 2014, the phenylacetaldehyde‐based lures (PHEN) attracted significantly more specimens than both the isoamyl alcohol‐based semisynthetic lures (SBL) and unbaited traps. SBL baits did not show a significant effect compared to the unbaited control. In 2015, the same trend could be seen but the difference was significant only between the two baits (PHEN and SBL; Figure 3).

In the case of *M. thalassinum*, SBL lure was significantly more efficient than the PHEN lures in both studied years. Because of relatively high catches of unbaited traps, the SBL lure could not attract significantly more individuals than empty traps in 2014. Contrary in 2015, when the number of total catches was higher the difference was significant (Figure 3).

The preferences of *C. fuscus* showed the same pattern as *M. thalassinum*. They were not affected by PHEN baits but showed a clear preference for SBL baits in both studied years. The absolute efficiency of SBL baits against *C. fuscus* could not be proved, because of relatively high catches of unbaited traps (Figure 3).

In the case of *R. nitidula*, the SBL baits attracted the most individuals, and the unbaited traps were the less effective, but these differences were not significant in any cases (Figure 3).

*Leptophyes albovittata* showed a clear preference to the phenylacetaldehyde‐based lures (PHEN), while the SBL lure had no influence on it in both years (Figure 3).

Considering *T. viridissima* and *P. griseoaptera* the total number of individuals caught was not enough for statistical analysis. *T. viridissima* was attracted by SBL baits, while *P. griseoaptera* was caught only in 2015 by both types of traps (Table 1).

### 3.2 Results of passive citizen science

During the web search, 307 photos of the seven studied species were found. In 189 photos (61.6%), insects are staying on flowers or feeding with them. In 149 pictures 70 plant species belonging to 25 families could be identified at species level (Table 2). In the other 40 photos plants could be identified only at family level.

**TABLE 2:** Main data of pictures (pics.) collected during the web search on flower visitation of seven Orthoptera species: number of pictures, plant families and species on the pictures and ratio of plant species (%) belonging the most frequent plant families by orthopterans and in the whole sample.

	*Meconema thalassinum*	*Tettigonia viridissima*	*Pholidoptera griseoaptera*	*Phaneroptera falcata*	*Leptophyes albovittata*	*Conocephalus fuscus*	*Ruspolia nitidula*	Sum
Total number of pics.	15	62	30	76	56	60	10	307
Number of pics. with flowers	4	31	12	59	44	32	7	189
Ratio of pics. with flowers (%)	26.7	50.0	40.0	77.6	78.6	53.3	70.0	61.6
Plant families	2	11	8	13	7	6	3	25
Plant species	2	21	7	25	19	10	5	70
Ratio of photos (%)								
Asteraceae	75.0	54.8	25.0	66.1	61.4	75.0	57.1	61.9
Apiaceae	0.0	0.0	8.3	6.8	20.5	3.1	14.3	8.5
Fabaceae	0.0	6.5	16.7	0.0	0.0	12.5	0.0	4.2
Rosaceae	25.0	6.5	0.0	0.0	6.8	0.0	0.0	3.2
Convolvulaceae	0.0	3.2	0.0	3.4	2.3	3.1	0.0	2.6
Others	0.0	29.0	50.0	23.7	9.0	6.3	28.6	19.6

Most photos could be found for *P. falcata* (76), *T. viridissima* (62), *C. fuscus* (60) and *L. albovittata* (56). The ratio of pictures showing the species on flowers was highest in case of *L. albovittata* (78.6%) that was followed by *P. falcata* (77.6%), *R nitidula* (70.0%) and *C. fuscus* (53.3%). In case of the other species, this ratio was equal or <50% (Table 2).

Based on pictures Asteraceae family was the most frequently documented with 22 species including the most visited *Tanacetum vulgare*, *Pulicaria dysenterica*, *Achillea millefolium*, *Solidago canadensis*, and *Centaurea scabiosa*. Each Orthoptera species has been photographed with a higher ratio on different Asteraceae species (Table 2).

Most photos of *P. falcata* were taken on three Asteraceae plants: *S. canadensis* (5), *T. vulgare* (4), *A. millefolium* (3), and also on *Daucus carota* (Apiaceae) (3). *L. albovittata* was mostly observed on *C. scabiosa* (4) and *Cirsium vulgare* (3) (Asteraceae), and *Salvia verticillata* (Lamiaceae) (4).

More than 30 photos with flowers were found both for *T. viridissima* and *C. fuscus*. Most *T. viridissima* specimens were photographed on Asteraceae plants as *A. millefolium* (3), *Echinacea purpurea* (3) and *T. vulgare* (2) and on *Lilium* × *hybridum* (2) belonging to the Liliaceae family. For the *C. fuscus*, the most frequent plants were *Pulicaria dysenterica* (9), *T. vulgare* (6) and *Tagetes patula* (3) belonging to the Asteraceae family and one Fabaceae species *Trifolium incarnatum* (3).

In the case of the other species, <15 photos with flowers per species were found. In the case of *P. griseoaptera*, *Achillea filipendula* (Asteraceae) (2), *Coronilla varia* (Fabaceae) (2), and *Orchis militaris* (Orchidaceae) (2) were the most frequent plants on the photos. Regarding to *R. nitidula*, more than half of the photos were taken on *Eupatorium cannabinum* (Asteraceae) (2) and *Lythrum salicaria* (Lythraceae) (2), while in the case of *M. thalassinum*, *Calendula officinalis* (Asteraceae) (2) and *Sanguisorba minor* (Rosaceae) (1) were the most frequent plants on the pictures.

## 4 DISCUSSION

Over our 2‐year study, seven Orthoptera species (*Phaneroptera falcata*, *Meconema thalassinum*, *Conocephalus fuscus*, *Ruspolia nitidula*, *Leptophyes albovittata*, *Tettigonia viridissima*, and *Pholidoptera griseoaptera*) were sampled by traps baited with synthetic phenylacetaldehyde‐based (PHEN) and semisynthetic isoamyl alcohol‐based baits (SBL) designed for monitoring of Lepidoptera pests. Each caught species is a common and sometimes abundant member of the local fauna, and they are all widely distributed eurytopic, Eurasian species (Heller et al., 1998; Nagy & Rácz, 2007; Szanyi et al., 2017).

Although each sampled species belongs to the thamnobiont life form (Nagy & Rácz, 2007), they have different habitat preferences. *M. thalassinum*, *T. viridissima*, and *P. griseoaptera* prefer shrubby vegetation of forest edges (Diekotter et al., 2005), and the first two species spend significant time in the canopy of shrubs and smaller trees where the traps were placed. *P. griseoaptera* uses the lower part of the vegetation of the same habitats. Contrarily, *C. fuscus*, *L. albovittata*, and *R. nitidula* are characteristic species of humid meadows and spend their lifespan in tall and dense grasses; moreover, *L. albovittata* often occupies flower‐rich grasses in mesic habitats too. *Phaneroptera falcata* shows intermediate character since their nymphs and young adults feed and inhabit grasses during the summer, and then from late August, when the nights become colder adults stay mainly in the canopy. Males stridulate in the higher parts of shrubs and females lay their eggs also into the leaves of these plants (Samietz et al., 2017).

Temperate zone tettigonoids are mainly mixophagous. They feed both on vegetative and generative parts of different host plants and predate small arthropods parallelly, but we know little about their host plant and prey preferences. Among the first “canopy‐living” group of species, *M. thalassinum* showed a significant preference to SBL bait. This is the first report on volatiles attractive to *M. thalassinum*, which help us to understand the its food preference. Harz (1957) reported that this small bush cricket is mainly carnivorous and predates small caterpillars and aphids living in the canopy and only secondarily feeds on green parts of the trees. Our result suggests that we should rethink our knowledge since the plant parts, which have a similar chemical character as our SBL lure (e.g. *Rosa* spp., *Prunus* spp.; pherobase.com), may have larger importance in its feeding.

The VARL+ traps used are designed for catching large lepidopterans such as Noctuidae and Pyralidae species; however, they are not efficient for as large insects as *T. viridissima*, and the position of the traps was far from the layer used by flightless *P. griseoaptera*, which can explain their low abundances in the traps. Both of them have been reported as omnivorous species mostly preying on caterpillars, aphids, small moths, and other small invertebrates (Harz, 1975; Lupu, 2007). Nevertheless, most of the individuals caught were trapped by SBL traps, suggesting that species living in forest edges have similar chemical and food preferences parallelly. The preference for SBL lure refers to the preference of these species for arboreal plants.

Unexpectedly, the grass‐living thamnobiont *C. fuscus* showed a similar preference for SBL lure, but it was significant only in comparison with PHENs baited traps. The effect of SBL is not clear, because of the relatively high catches of control traps. Although *C. fuscus* is a mesopterous species, they are not good‐flyers, so they should track to the traps through branches of the trees and bushes, which suggests the larger attractiveness of SBL lure than the mean number of caught individuals showed. Parallelly, the habitat preference of this species may also need reconsideration.

*Ruspolia nitidula* has the same habitat preference as *C. fuscus*, and it was also attracted by the SBL lure, but in this case, the PHENs have a lower effect. Since there were no significant differences, the exploration of the effect of the lures on these two species needs further investigation.

In the case of *L. albovittata* Harz (1957) and Marini et al. (2009) reported that they like to stay on shrubs such as blackthorn (*Prunus spinosa* L.), bramble (*Rubus* spp.) and blueberry (*Vaccinium* spp.) and often feed on leaves and flowers of pungent or aromatic plants as mint (*Mentha* spp.), sage (*Salvia* spp.), nettle (*Urtica* spp.) and dead‐nettle (*Lamium* spp.). The catches showed the attractivity of PHEN lures containing phenylacetaldehyde, (*E*)‐anethol, eugenol, and benzyl acetate, which is the first report on the allelochemicals that attract *L. albovittata*. Since data on whether the mentioned host plants contain any of the compounds of the tested baits were not found, the evaluation of the host plant preference of *L. albovittata* needs further detailed investigations.

*Phaneroptera brevis* is one of the few known floriphilic Orthoptera species. It lives in tropical South‐East Asia (Strother & Weedon, 2006), while in Hungary there are two *Phaneroptera* species (*P. falcata* and *P. nana* Fieber) can be found. They are omnivorous species (Harz, 1960) without any detailed data on their host plants in Europe. Here, we provide the first data on the attractivity of PHEN lures in the temperate zone *Phaneroptera* species *P. falcata*. Compounds of PHEN lures are widely distributed components of flower scents and one or more of them may be found in numerous herbaceous plant species belonging to different families (pherobase.com), so the evaluation of the detailed preference of *P. falcata* needs further investigations. In extremely xeric weather conditions of 2022, when surrounding natural grasses dried, a large number of *P. falcata* nymphs feed on flowers of irrigated pelargoniums (*Pelargonium* spp.), carnations (*Dianthus* spp.) and roses (*Rosa* spp.) in the suburban region of Debrecen (East Hungary) (A. Nagy, unpublished data). These plant species contain one or more compounds of PHEN baits that also support our findings (Jürgens et al., 2003).

In the passive citizen science study, large ratio of *P. falcata* and *L. albovittata* photos show these species on flowers, which refer to the flower‐visiting habit of them, that support our findings. In the case of *P. falcata* one of the preferred plants *Achillea millefolium* contains eugenol (Anderson, 2003; Shawl et al., 2002), while on some pictures, this bush cricket could be seen on *Syringa vulgaris* containing phenylacetaldehyde (Brunke et al., 1992; Mookherjee et al., 1990) and *Mirabilis jalapa* containing eugenol, phenylacetaldehyde, and benzyl acetate together (Levin et al., 2001).

Based on photos, *L. albovittata* prefers Asteraceae, Apiaceae, and Rosaceae species, and many species of these family contain one or more compounds of PHEN lures (phenylacetaldehyde: *Cirsium arvense*, *Eupatorium cannabinum* (Anderson, 2003), eugenol: *Achillea millefolium* (Shawl et al., 2002), *Helianthus annuus* (Pham‐Delegue et al., 1989); benzyl‐acetate: *Senecio articulates* (Kite & Smith, 1997)). In the case of other plant species appearing in the photos, the presence of the compounds is not known.

These results show that our knowledge of the host plant and habitat preference of these relatively well‐known species needs revision. Further investigations can clarify the feeding habits and ecological requirements of these and other related species and help to understand their role in local ecosystems.

## AUTHOR CONTRIBUTIONS

**Antal Nagy:** Conceptualization (lead); data curation (equal); formal analysis (lead); investigation (equal); methodology (equal); software (equal); writing – original draft (equal); writing – review and editing (equal). **Aletta Ősz:** Conceptualization (equal); data curation (equal); investigation (equal); methodology (equal); writing – original draft (equal); writing – review and editing (equal). **Miklós Tóth:** Conceptualization (equal); formal analysis (equal); funding acquisition (equal); methodology (equal); writing – original draft (equal); writing – review and editing (equal). **István András Rácz:** Conceptualization (equal); investigation (equal); methodology (equal); writing – original draft (equal); writing – review and editing (equal). **Szilvia Kovács:** Conceptualization (equal); investigation (equal); writing – original draft (equal). **Szabolcs Szanyi:** Conceptualization (lead); data curation (equal); funding acquisition (equal); investigation (lead); methodology (equal); writing – original draft (equal).

## CONFLICT OF INTEREST STATEMENT

The authors declare no competing interests.

## Data Availability

The datasets generated during and/or analyzed during the current study are available in the ZENODO repository, Nagy et al. (2022). Nontarget catches of traps with chemical lures may refer to the flower‐visitation, probable pollination, and feeding of bush crickets (Ensifera: Tettigoniidae). https://doi.org/10.5281/zenodo.7130653.
